# Insights into the mechanism underlying feather pattern formation of sex-linked barring in Chinese native chickens

**DOI:** 10.1016/j.psj.2026.106945

**Published:** 2026-04-16

**Authors:** Qian Xue, Guohui Li, Huiyong Zhang, Jianmei Yin, Chenghao Zhou, Yixiu Jiang, Xing Ju, Wei Han

**Affiliations:** a Jiangsu Institute of Poultry Science, Yangzhou 225125, China; b Technology Innovation Co., Ltd., Jiangsu Institute of Poultry Science, Yangzhou 225125, China; c Key Laboratory for Evaluation and Utilization of Livestock and Poultry Resources (Poultry), Ministry of Agriculture and Rural Affairs, P.R. 225125, China

**Keywords:** Transcriptome, Sex-linked barred feather, Expression pattern, Molecular regulation, Chinese native chicken

## Abstract

The sex-linked barring of chicken feather pattern is a fascinating trait, and it has great application value in chicken breeding by enabling autosexing. In this study, the transcriptome changes in skin follicle tissues from the back (SKs) and crown of the head (SKHs) were explored by RNA-seq in Wenshang Barred chickens during their feather pattern formation. The key genes and signaling pathways regulating sex-linked barring formation in chickens were analyzed. As a result, a total of 2291 and 4496 differentially expressed genes (DEGs) were found in SKs and SKHs, respectively, during barred feather formation. The expression pattern clusters of Profile 6 and Profile 7 were the important and mainstream expression trends of the DEGs. Melanogenesis KEGG pathway and GO terms of melanocyte differentiation, melanosome organization and melanin biosynthetic biological processes were significantly enriched in the DEGs, including the well-known TYR, TYRP1, EDNRB and PMEL genes, clustered in Profile 6 expression pattern. A series of DEGs, including CDKN2A, SFN, CDKN2B and CDK1, were significantly enriched in the cell cycle pathway and were mostly clustered in Profile 7. Protein‒protein interaction (PPI) network analysis revealed TYR, TYRP1, CDKN2A and SFN were the hub genes. It was speculated that CDKN2A may target SFN to regulate the melanocyte cycle arrest, causing the formation of white bands of sex-linked barring. TYR, TYRP1, EDNRB and PMEL genes may play important regulatory roles by melanogenesis pathway in the early growth of black feathers, as well as in the later formation of black stripes. The different expression patterns of SLC45A2, KIT, MC1R, ASIP, DCT and SOX10 in SKs and SKHs may contribute to the different formation processes of barred feather patterns on the head and the back. These results provide new insights into the regulation of sex-linked barred feather patterns in Chinese native chicken and provide a valuable theoretical foundation for future chicken breeding and related efforts.

## Introduction

The plumage colors and patterns of domestic chickens are rich and diverse. In particular, in China, the breeds and genetic resources of local chickens are abundant, and most breeds have a specific plumage color and pattern. Common plumage colors in domestic chickens include yellow, black, white, barred and speckled feathers. The feather color of chickens is an important part of their physical features. A thorough understanding of the molecular mechanisms governing feather color formation is essential not only for developing distinct color varieties through breeding but also for identifying and distinguishing local breeds.

Barred plumage is a typical feather color in domestic chickens. There are two different barring patterns in chickens: autosomal and sex-linked barring. Both barring patterns are characterized by alternating bars of two different colors on individual feathers. However, it has been reported that chickens carrying autosomal barring exhibit a black bar on a white or red background, while the feathers of sex-linked barred chickens are characterized by a white bar on a red or black background (Doreen et al., 2017). Autosomal barring patterns are irregular and slightly messy, whereas sex-linked barring patterns are more regular. Other characteristics of sex-linked barring are the dilution of dermal pigment in the shanks and beak as well as a white spot on the head present at hatch, which can be utilized for early sex determination (Jerome, 1939). The mechanisms responsible for the formation of these two barring patterns differ. In sex-linked barring, the nonblack sections of the barred pattern result from the absence of melanocytes capable of producing melanin. Conversely, in autosomal barring, the nonblack portions of the barred pattern arise because of the inhibition of melanin production rather than the lack of melanocytes (Doreen et al., 2017; Yang et al., 2019; Andersson et al., 2020).

Most of the past studies have mainly focused on the localization of genes and identification of loci of chicken sex-linked barring (Spillman, 1908; Bitgood, 1988; Dorshorst et al., 2009). In 2010, the dominant alleles associated with sex-linked barring were determined to be located to CDKN2A/B loci on Z chromosome. Four SNPs were identified within *CDKN2A* with two non-coding mutations respectively in two introns and two missense mutations both in exon 1 (Anders et al., 2010). In 2017, a further exploration (Doreen et al., 2017) showed that the four mutations were all involved in the sex-linked barring of Barred Plymouth Rock chicken. Chinese researcher have also investigated Chinese native chickens with barring plumage, and only 3 SNPs (SNP1, SNP2 and SNP3) in CDKN2A have been identified (Fu, 2019). Recently, the sex-linked barring feather gene can be isolated from White Leghorn chickens, and only SNP4 in CDKN2A was applicable for barring gene identification in this population (Cai et al., 2025). Therefore, CDKN2A was evaluated as a major locus of sex-linked barring feather, although the functional loci in it may vary in different population. However, there are few reports on the specific molecular processes regulating sex-linked barring in Chinese native chicken. Other genes interacting with CDKN2A and the key signaling pathways involved in the formation of chicken sex-linked barring patterns remain unclear.

**Fig. 1:**
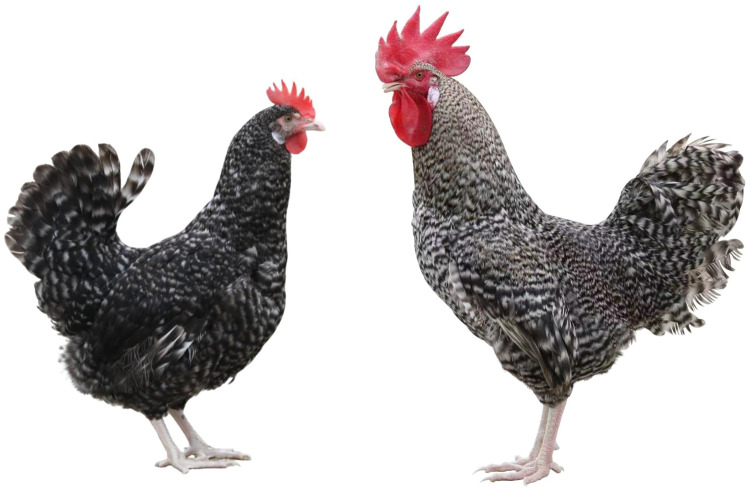
Wenshang Barred chicken.

Chinese native chickens with a barring phenotype include Wenshang Barred chickens, Wenchang chickens, Tibetan chickens, and Hongguang Barred chickens. Among them, Wenshang Barred chickens have evenly distributed black and white striped feathers all over their body. There is a white spot on the head at hatch. It is the only local chicken breed in China that is characterized by sex-linked barring feathers (Niu et al., 2024). The Wenshang Barred chicken breed originated in Wenshang County, Shandong Province, China. This breed has a long history and it has been recognized for its excellent egg-laying performance, desirable carcass traits, and high meat quality; thus, it is classified as both a dual-purpose and ornamental breed (Yang et al., 2022). In particular, its sex-linked barring trait could be used to develop a new chicken breed (or strain), which can be highly convenient for chicken production by enabling autosexing. Therefore, Wenshang Barred chicken is an excellent material for chicken breeding (Fig. 1). In this study, Wenshang Barred chickens were used as the experimental subjects, the changes in the transcriptome of skin with follicles during the formation of whole-body barred feathers were analyzed, and the regulatory mechanism underlying the formation of sex-linked barring patterns in Chinese native chickens was explored.

## Materials and methods

### Ethics statement

All experimental procedures involving chickens were performed in accordance with the Regulations for The Administration of Affairs Concerning Experimental Animals approved by the State Council of the People’s Republic of China. Moreover, this study was approved by the Animal Experiments Ethics Committee of Jiangsu Institute of Poultry Science under permit number JIPSAEC2022-087.

### Animal and tissue sampling

Wenshang Barred chickens were raised and conserved in the National Gene Bank of Local Chicken Breeds (Jiangsu, China). During their growth process, the changes in their feather color were observed, and three representative stages were focused on. When the chicks had just hatched, black villi were distributed throughout the body, except for white spots on the head. At approximately 2 weeks of age, black and white striped feathers appeared just on the wings. At 5 weeks of age, the barred feather patterns had completely formed all over the body. A total of 18 females, 6 from each of the three stages, were selected and euthanized. The skin tissues with complete and abundant hair follicles retained were collected from the same location of the back (center of the dorsal) and the head (crown of the head). Each of the skin sample approximately 1.0 × 1.0 cm^2^ in size was taken and washed with Phosphate-Buffered Saline (PBS). Afterward, they were immediately frozen in liquid nitrogen and stored at −80°C for the subsequent extraction of total RNA.

### Total RNA extraction, library construction and sequencing

Total RNA was extracted from each of the 36 tissue samples using TRIzol (Invitrogen, CA, USA). RNA purity and integrity were monitored by a NanoDrop 2000 spectrophotometer (NanoDrop Technologies, Wilmington, DE, USA) and a Bioanalyzer 2100 system (Agilent Technologies, CA, USA). The purity of RNA samples met to standards when the OD260/OD280 ratio fell within the range of 1.8 - 2.1, and the OD260/OD230 ratio exceeded 2.0. The integrity should meet the requirement that the value of RNA Integrity Number (RIN) is no less than 7.0. Concurrently, RNA contamination was assessed by 1.5% agarose gel electrophoresis. Oligo(dT)-attached magnetic beads were used to purify mRNA. The purified mRNA was fragmented into small pieces with fragmentation buffer at the appropriate temperature. First-strand cDNA was subsequently generated using random hexamer-primed reverse transcription, followed by second-strand cDNA synthesis and purification using AMPure XP Beads. Afterward, A-Tailing Mix and RNA Index Adapters were added to end cDNA repair. The cDNA fragments obtained from the previous steps were amplified by PCR, and the products were subsequently purified by Ampure XP Bead to obtain the final library. The qualified library was used for RNA-seq on the MGISEQ-2000 platform (BGI, Shenzhen, China) to generate 150 bp paired-end reads.

### Quality control of sequencing data and gene expression detection

SOAPnuke (V2.1.0) (Chen et al., 2018) was used to filter the sequencing data as follows: (1) reads containing sequencing joints were removed; (2) reads with N ratios greater than 0.5% were removed; and (3) low-quality reads (those with Qphred ≤ 20 bases accounting for more than 50% of the length of the entire read) were removed. After filtering, clean reads were obtained, which were mapped to the GRCg7b reference genome using HISAT2.0.4 software (Kim et al., 2015). RSEM (V1.3.1) (Li et al., 2011) was used to calculate the number of reads aligned to each transcript, which was converted to fragments per kilobase per million bases (FPKM), thereby yielding the expression levels of genes and transcripts for each sample.

### Screening of differentially expressed genes (DEGs) and functional enrichment analysis

DESeq2 (V1.22.2) was used for differential expression significance analysis, and the screening criteria were an FDR (false discovery rate) < 0.05 and a log2FC (fold change) > 1 or < −1. On the basis of the GO and KEGG databases, all the detected genes were subjected to functional annotation. Enrichment analysis of the DEGs was performed by using the DAVID online platform (Sherman et al., 2022). GO terms and KEGG pathways with adjusted P values < 0.05 were considered significant.

### Gene expression pattern analysis and clustering

The gene expression pattern was analyzed by Short Timeseries Expression Miner (STEM) software (V1.3.12) (Jason et al., 2006). The ‘log normalized data’ method was adopted in the strategy of expression quantity transformation. Other options were set to default since they have been shown to yield optimal results with both biological and simulated data (Jason et al., 2005). The expression level used in this study was the previously calculated FPKM value. The *P* value of the clustered profile was less than 0.05, which was considered significant (Xu et al., 2018). By applying the above methods, the gene expression patterns of skin follicle tissues from the back and crown of the head of Wenshang Barred chickens were clustered.

### Protein‒protein interaction (PPI) network analysis for DEGs

PPI network analysis of the screened DEGs was performed using STRING database software (http://string-db.org/). The interacting protein pairs were imported into Cytoscape 3.10 software for visualization. By using the CytoHubba plugin in this software (Chin et al., 2014), several algorithms, such as MCC, MNC, Degree, EPC and DMNC, were employed to analyze and screen the hub genes in the network.

### Validation of RNA-Seq Data

To verify the accuracy of RNA-seq data and analysis results, quantitative Real-Time PCR (qPCR) technology was used to detect expression levels of the selected candidate genes in the same samples. The primers are shown in Table 1. The cDNA was generated by reverse transcription using HiScript II qRT SuperMix (Vazyme, China) according to the manufacturer’s guidelines. Then, the qRT-PCR was performed in a volume of 20 μL for each reaction, containing 10 μL of 2 × SYBR Green Master Mix (Vazyme, China), 3 μL cDNA template, 0.8 μL of each primer (10 μmol) and 5.4 μL distilled deionized water. The following reaction conditions were used: denaturation at 95°C for 30 s; 40 amplification cycles of 95°C for 15 s, annealing temperature at 59 ∼ 61°C for 30 s; followed by a single melt cycle of 95°C for 15 s and 65°C for 1 min. The results were normalized to *β-ACTIN* expression. Relative expression of each gene was determined using the 2^-∆∆Ct^ method. Pearson’s correlation coefficient (R) was further calculated for each gene and Student’s t tests were used to quantify the consistency between the results of RNA-seq and qRT-PCR. P < 0.05 and P < 0.01 were considered significant and highly significant, respectively.

**Table 1:** Information of the primers used for qPCR.

Gene	Primers (5′−3′)	Size (bp)	TM (°C)
TYRP1	F:CAACATGGTGCCTTTCTGGC	101 bp	60
	R:CCCTACCTGGCCACTCAATG		
CDKN2A	F:GAAGCGCGGAAGAAGACACC	123 bp	61
	R:GGCAACCGACGGAATGTTTG		
SLC45A2	F:TTCACGGATTTCATGGGGCA	126 bp	60
	R:AATCGCATTGATGCACAGCC		
SOX10	F:CCCTGGACCGTAAGAGGAGA	111 bp	60
	R:GCAGCTGACAAAGAGTTGGC		
SFN	F: AGGAGGGCGACGACAAAG	125 bp	59
	R:CAGGTATCGGAAGTAGTCTCCC		
β-ACTIN	F:CGGACTGTTACCAACACCCA	115 bp	60
	R:TCCTGAGTCAAGCGCCAAAA		

## Results

### Changes of the feather color and patterns in Wenshang Barred chickens

**Fig. 2:**
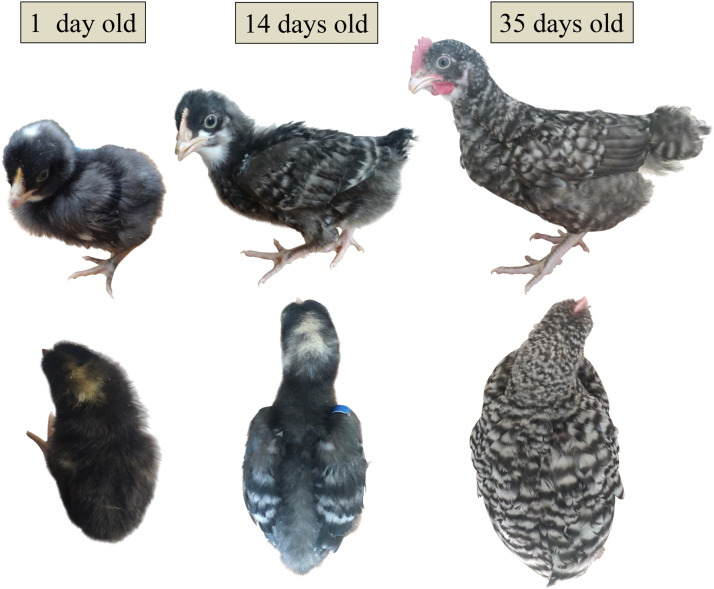
Changes in feather patterns of Wenshang Barred chickens at different stages. Corresponding to different age, the above image is a side view and the below one is a top-down view.

Changes in the feather color of Wenshang Barred chickens were observed from birth until the complete formation of barred plumage patterns all over the body. It was found that the newborn chicks have black downy feathers covering most of their bodies, with white spots of varying sizes on their heads. Some of the chicks also have a few white hairs on the tips of their wings or tails and under their lower jaws. White head spots are the most typical feature. As the chicks grow, the head spots seem to expand gradually, but their color appears to darken. The feathers on their bodies seem to gradually dilute from black to gray, resulting in a paler color. At approximately two weeks of age, the wings of the chicks show distinct black and white horizontal stripes; however, no obvious white stripes appear on any other parts of the body. At 5 weeks of age, the white head spots disappear, and the entire body, including the neck, abdomen, back and tail, is covered with an even distribution of black and white horizontal stripes. The sex-linked barred patterns were completely formed and fixed (Fig. 2).

### Statistics of the sequencing results

After quality control, an average of approximately 32.52 M and 31.23 M clean reads were obtained for the skin follicle tissues from the back and head, respectively. On average, 9.76 Gb of valid data were obtained from each skin follicle tissue from the back (SKs), and 9.37 Gb of valid data were obtained from each skin follicle tissue sample from the head (SKHs). The base mass values Q30 were all above 90%. The GC content of the clean reads ranged from 48.04% to 48.72%. Overall, the sequencing data met the requirements for subsequent analysis (Table 2).

**Table 2:** Summary of the sequencing data.

Tissues	Sampling ages (day)	Sample names	Replicates	Average clean reads	Average valid data (Gb)	Q30 (%)	GC (%)
Skin follicle tissues from the back (SKs)	1	SK1	6	32,161,100	9.65	>90.64	>48.62
	14	SK2	6	32,856,172	9.86	>90.04	>48.72
	35	SK5	6	32,551,135	9.77	>90.36	>48.40
Skin follicle tissues from the head (SKHs)	1	SKH0	6	28,626,828	8.59	>92.42	>48.22
	14	SKH2	6	29,625,297	8.89	>94.41	>48.04
	35	SKH5	6	35,438,031	10.63	>94.72	>48.44

### DEG identification during the formation of sex-linked barring pattern

**Fig. 3:**
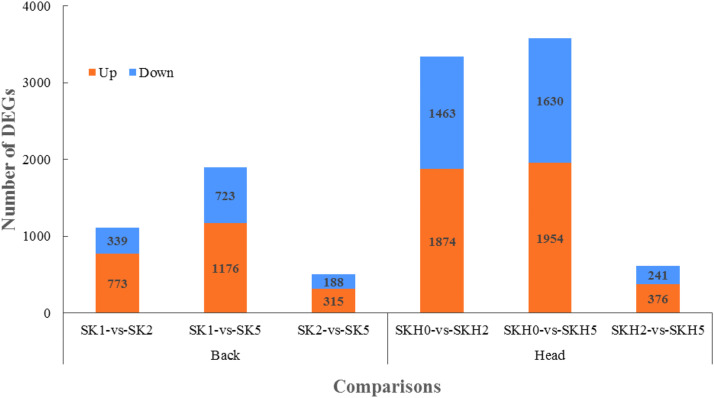
Distribution of the number of DEGs in each comparison group.

**Fig. 4:**
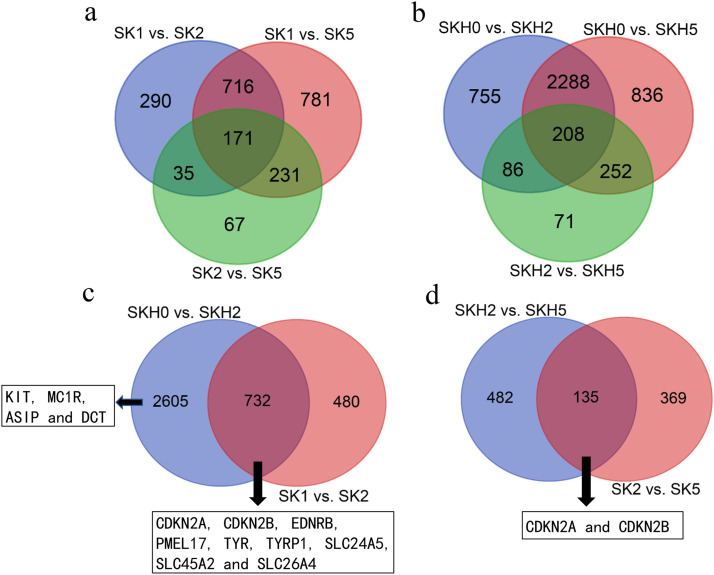
Venn diagrams of common DEGs among the comparisons.

Differences in gene expression at three stages during barred feather formation were examined in the two skin follicle tissues, and DEGs were identified by pairwise comparisons (Fig. 3, Supplementary Fig. 1). Comparisons of the three stages in SKs revealed 1212, 1899, and 504 DEGs in pairs of SK1 vs. SK2, SK1 vs. SK5, and SK2 vs. SK5, respectively. Comparisons of the three stages in SKHs revealed 3337, 3584, and 617 DEGs in pairs of SKH0 vs. SKH2, SKH0 vs. SKH5, and SKH2 vs. SKH5, respectively. The number of upregulated DEGs in each comparison was greater than that of downregulated DEGs. In the comparison between any two stages, the total number of DEGs in SKHs was much greater than that in SKs. In total, 2291 and 4496 genes were differentially expressed in SKs and SKHs, respectively, during barred feather formation. In addition, 171 and 208 genes were found to be the common DEGs of the three comparisons in SKs and SKHs, respectively (Fig. 4a and b). From 1 to 14 days of age, 732 genes were differentially expressed in both SKs and SKHs (Fig. 4c, Supplementary Table 1), including some widely reported feather pigmentation-related genes (EDNRB, PMEL, TYR, and TYRP1) and some solute carrier family genes (SLC24A5, SLC45A2, and SLC26A4). CDKN2A and CDKN2B, two cell cycle genes, were also included. Four genes associated with melanin production (KIT, MC1R, ASIP and DCT) were found to be differentially expressed only in the comparison of SKH0 vs. SKH2 but not in SK1 vs. SK2. From 14 to 35 days of age, 135 genes were differentially expressed in both SKs and SKHs (Fig. 4d, Supplementary Table 2). Among them, two cell cycle-related genes, CDKN2A and CDKN2B, were included, but no other reported melanin-related genes were found. Therefore, CDKN2A and CDKN2B may play major roles in the formation of the barred pattern on feathers over the entire body.

### Gene expression profile clusters during sex-linked barring pattern formation

**Fig. 5:**
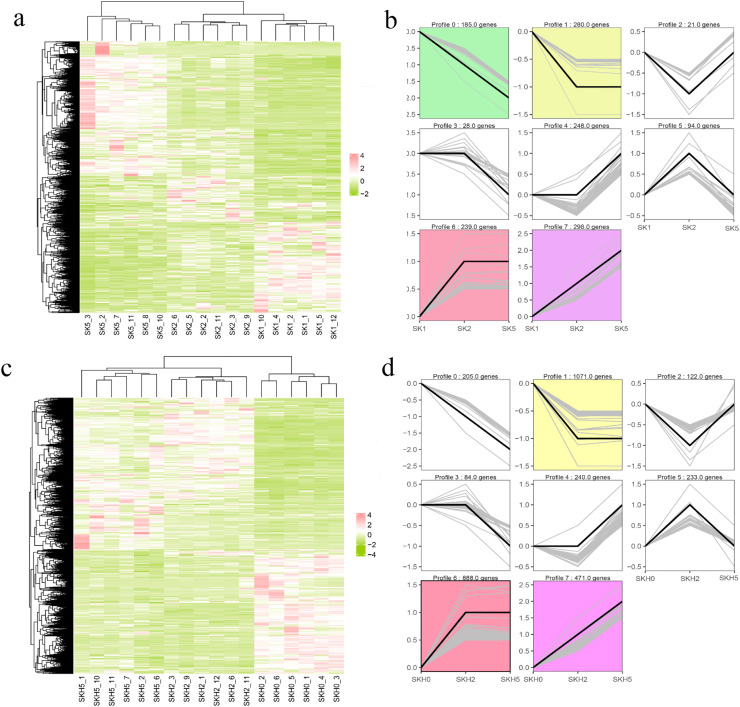
Heatmaps of DEGs across the three stages and clusters of different expression patterns. (a and c) Heatmaps of DEGs in SKs (a) and SKHs (c). The colors from olive to white to pink represent low, medium and high expression levels, respectively. (b and d) Expression pattern clustering analysis of DEGs in SKs (b) and SKHs (d). The colored profile clusters are the significant ones with P < 0.05.

Hierarchical clustering of all the DEGs in SKs and SKHs was performed according to their expression levels (FPKM values). The results revealed that the replicates from the same groups clustered together (Fig. 5a and c), confirming the accuracy and reliability of the collected samples and the sequencing data. Clustering of expression profiles was conducted to identify DEGs with the same expression profiles and to analyze the important and mainstream expression trends of DEGs during the formation of the barred feather pattern. The results from 2291 DEGs in SKs revealed that four expression profiles were significantly clustered (P < 0.05), namely, Profile 0, Profile 1, Profile 6 and Profile 7 (Fig. 5b). The Profile 0 cluster contained 185 genes whose expression continuously decreased throughout the entire process. These genes included SOX10, known to be involved in melanocyte development. The 280 genes in the Profile 1 cluster were significantly downregulated from hatching to 14 days of age and remained almost unchanged from 14 days to 35 days of age. There were 239 genes in Profile 6. The expression levels of these genes increased at 14 days of age but remained unchanged until 35 days of age. Genes involved in melanin synthesis, such as TYR, TYRP1, PMEL, EDNRB and SLC24A5, belonged to this cluster. The Profile 7 cluster had the greatest number of DEGs (298), whose expression pattern was opposite that of the Profile 0 cluster; their expression level continued to increase from hatching to 35 days. The CDKN2A gene, reported to be associated with sex-linked barring, was included in this cluster.

A cluster analysis of the expression patterns of 4,496 DEGs in SKHs was also performed, and three significant clusters were found (P < 0.05), namely, Profile 1, Profile 6 and Profile 7 (Fig. 5d). These three clusters exhibited the same expression profiles as the corresponding clusters in SKs. The Profile 1 cluster contained the greatest number of genes, 1071. Among the 888 genes in the Profile 6 cluster, KIT, ASIP, and SLC45A2 are related to plumage color. The Profile 7 cluster included 471 genes, including CDKN2B, associated with the cell cycle.

### Functional annotation and enrichment analysis of DEGs in different clusters

**Fig. 6:**
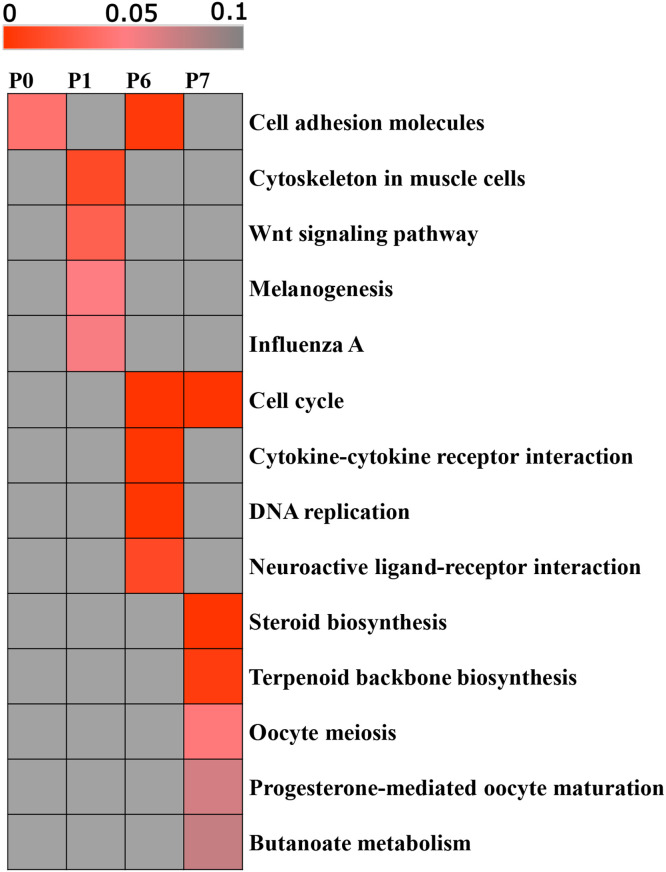
KEGG pathway enrichment analysis of DEGs in SKs with different profiles. The significance of the most enriched pathways in each main profile cluster is indicated by the P value (red). The dark gray areas represent the missing values.

To explore and analyze the functions of the DEGs in different expression profile clusters, GO and KEGG functional annotation and enrichment analyses were conducted on the DEGs in the significant clusters of the two tissues. The results revealed that in SKs, the DEGs in the four significantly clustered expression profiles of Profile 0, Profile 1, Profile 6 and Profile 7 were significantly enriched in 10, 30, 40 and 23 biological process GO terms, respectively (P < 0.05). Among the significantly enriched GO biological process terms of the Profile 6 and Profile 7 clusters, melanin biosynthetic process, melanosome organization, melanocyte differentiation, cell cycle and apoptotic process were included (Table 3, Supplementary Table 3). KEGG enrichment analysis revealed that DEGs in the Profile 0 cluster were significantly enriched in the cell adhesion molecule pathway. The DEGs in Profile 1 were significantly enriched in the cytoskeleton in muscle cells and Wnt signaling pathways. In addition, among the DEGs in Profile 1, five genes, namely, FZD2, WNT11, WNT5A, ADCY3 and WNT2, were enriched in the melanogenesis signaling pathway (Table 3). The DEGs in the Profile 6 and Profile 7 clusters were significantly enriched in 5 and 4 pathways, respectively (P < 0.05), among which the cell cycle was the most commonly enriched pathway, although the genes from the two clusters exhibited different expression patterns (Fig. 6).

**Table 3:** DEGs enriched in melanin-related biological processes and pathways with different expression patterns in SKs.

Cluster	Biological process GO terms/KEGG pathways	Gene count	P value	Genes
Profile 1	Melanogenesis pathway	5	0.05	FZD2, WNT11, WNT5A, ADCY3, WNT2
Profile 6	Melanin biosynthetic process	3	0.002	OCA2, TYRP1, TYR
	Melanosome organization	3	0.003	PMEL, GPR143, TYRP1
	Melanocyte differentiation	3	0.004	OCA2, TYRP1, SLC24A5

In SKHs, the DEGs in the three significantly clustered expression profiles of Profile 1, Profile 6 and Profile 7 were significantly enriched in 80, 38 and 41 GO biological process items, respectively (P < 0.05), involving multiple cell differentiation- and migration-related entries (Supplementary Table 4). KEGG enrichment analysis revealed that the DEGs in the Profile 1, Profile 6 and Profile 7 clusters were significantly enriched in 10, 12 and 8 signaling pathways, respectively. Most of these pathways were associated with growth and development, reproduction and metabolism. For example, the MAPK signaling pathway, TGF-β signaling pathway and focal adhesion pathway were significantly enriched among the DEGs in the Profile 1 cluster. The progesterone-mediated oocyte maturation and oocyte meiosis pathways were significantly enriched among the DEGs in the Profile 6 cluster. Anabolism metabolism-related pathways, such as sphingolipid, fatty acid and carbon metabolism, were significantly enriched among the DEGs in the Profile 7 cluster (Supplementary Table 5).

### Functional enrichment analysis of all DEGs during barred feather pattern formation

**Fig. 7:**
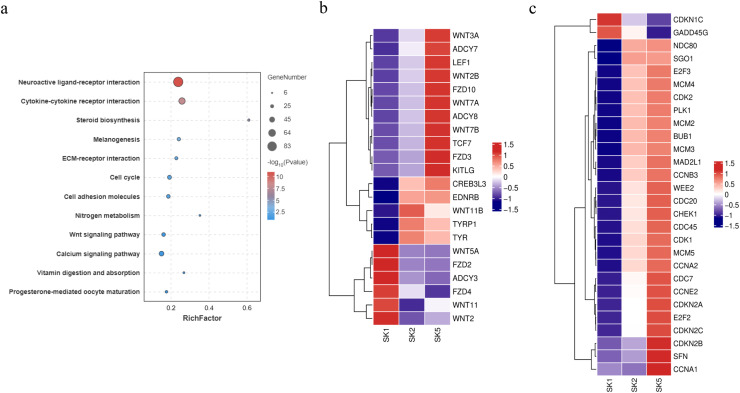
KEGG pathways enriched by DEGs in SKs (a) and expression heatmaps of 22 and 28 DEGs assigned to melanogenesis (b) and cell cycle (c) pathways, respectively, at three stages. The log-transformed expression values ranged from −1.5 to 1.5. Red and blue indicate up- and downregulated genes, respectively.

GO and KEGG enrichment analyses were performed on all the DEGs in the two tissues during barred feather pattern formation. In SKs, 2291 DEGs were significantly enriched in 42 biological process GO terms (P < 0.01), which involved growth and development, signal transduction and immunity, such as the Wnt signaling pathway, immune response and chemical synaptic transmission. Notably, a GO term of melanocyte differentiation was enriched by four DEGs, OCA2, TYRP1, BCL2 and SLC24A5 (Supplementary Table 6). KEGG enrichment analysis revealed that 8 signaling pathways, including neuroactive ligand–receptor interactions, cytokine–cytokine receptor interactions, steroid biosynthesis, melanogenesis and the cell cycle, were significantly enriched (P < 0.05) (Fig. 7a, Supplementary Table 7). Melanogenesis and cell cycle pathways, involving 22 and 28 DEGs, respectively, may be associated with the formation of barred plumage color (Fig. 7b and c).

**Fig. 8:**
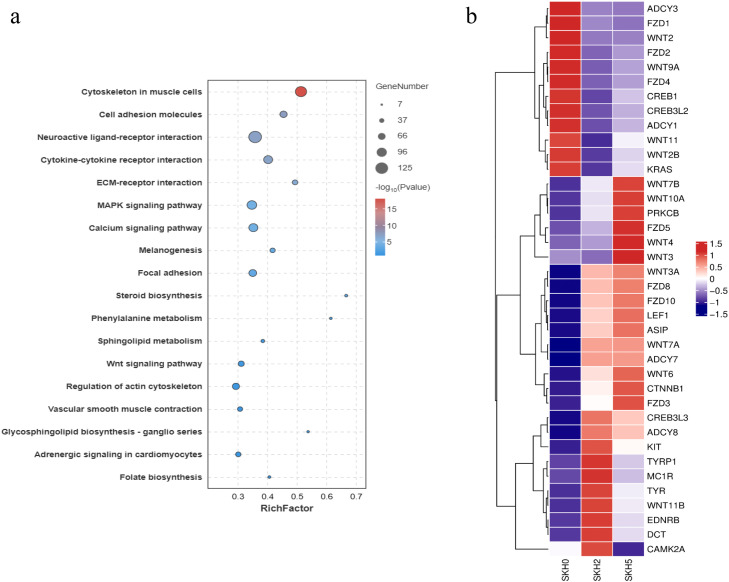
KEGG pathways enriched by DEGs in SKHs (a) and expression heatmap of 33 DEGs assigned to the melanogenesis pathway at three stages (b). The log-transformed expression values ranged from −1.5 to 1.5. Red and blue indicate up- and downregulated genes, respectively.

In SKHs, 4496 DEGs were found to be significantly enriched in 82 biological process GO terms (P < 0.01), with the top three terms being multicellular organism development, extracellular matrix organization and cell adhesion. Interestingly, the six DEGs LRMDA, OCA2, KIT, TYRP1, BCL2 and SLC24A5 were enriched in melanocyte differentiation GO term. The biological process term “melanosome organization” was enriched in 5 DEGs, namely, TYRP1, RAB32, PMEL, GPR143 and ASIP (Supplementary Table 8). KEGG enrichment analysis revealed that a total of 14 signaling pathways, including the cytoskeleton in muscle cells, cell adhesion molecules, neuroactive ligand–receptor interactions and melanogenesis, were significantly enriched (P < 0.05) (Fig. 8a, Supplementary Table 9). Among them, the melanogenesis pathway contained 38 DEGs, including ASIP, TYRP1, EDNRB, TYR, CREB3L3, CREB3L2, MC1R, DCT, KIT and other feather color-related genes (Fig. 8b).

### PPI network analysis of the related DEGs

**Fig. 9:**
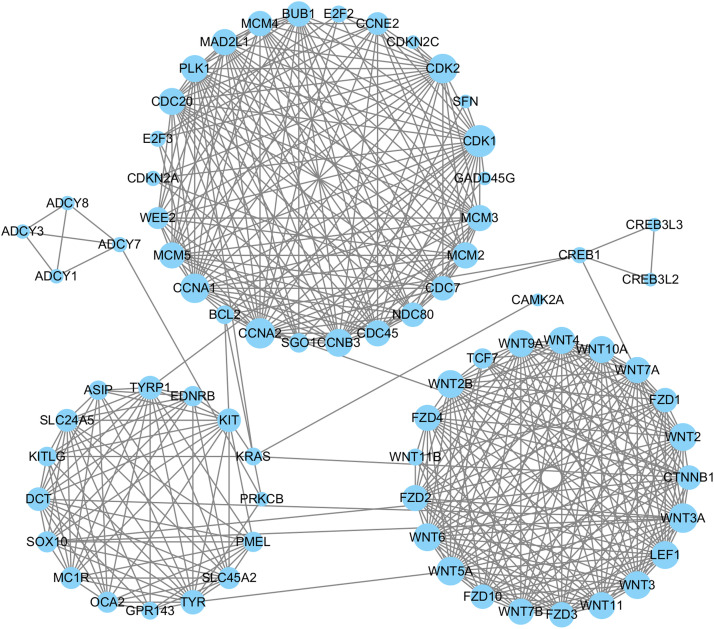
PPI networks of DEGs involved in melanogenesis and cell cycle pathways and related biological processes. Node size indicates connectivity.

**Fig. 10:**
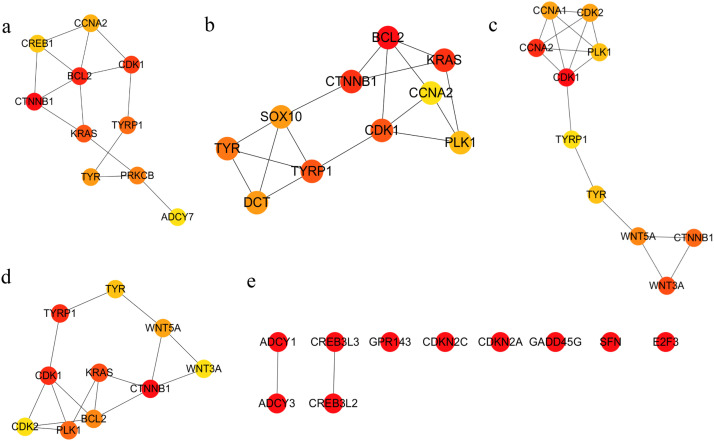
The top 10 hub genes of different algorithms. (a–e) The top 10 hub genes obtained by the 5 algorithms for the betweenness, radiality, closeness, bottom neck and clustering coefficients, respectively.

**Fig. 11:**
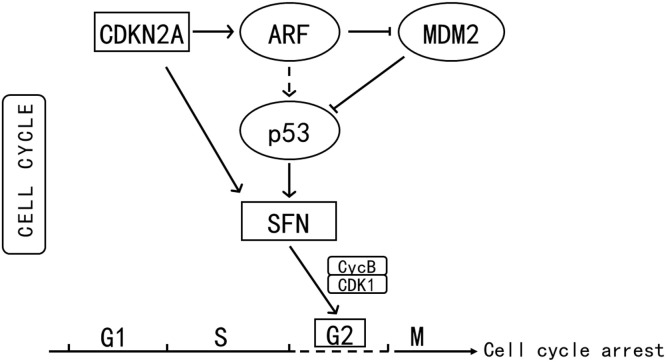
Diagram of the role of CDKN2A and SFN in the cell cycle pathway.

On the basis of the STRING online database, DEGs associated with the biological processes of melanin synthesis, melanocyte differentiation and melanosome organization and those involved in melanogenesis and cell cycle pathways were used for PPI network analysis. A network composed of 71 nodes (genes) and 450 edges (relationships) was constructed and visualized with Cytoscape 3.10 software (Fig. 9). The hub genes in the network were identified by using 12 algorithms, such as MNC, EcCentricity, BottleNeck and others, in the CytoHubba plugin of Cytoscape 3.10 software. TYR, TYRP1 and CDK1 were found to be common hub genes according to the results of multiple algorithms (Fig. 10a–d). Clustering coefficient algorithm analysis revealed that CDKN2A, SFN, GADD45G and other genes were important hub genes (Fig. 10e). These hub genes were inferred to play important roles in the regulatory network. Pathway maps from the KEGG database revealed that CDKN2A and SFN are genes that participate in the cell cycle and p53 signaling pathways (Supplementary Figs. 2 and 3). SFN is the downstream target in these two pathways and induces cell cycle arrest at the G2 phase by inhibiting the CycB/CDK1 protein complex (Kanehisa et al., 2023) (Fig. 11). Therefore, CDKN2A and SFN may play key roles in the regulation of feather pattern formation through the cell cycle pathway.

### Expression patterns of the candidate genes and validations by qPCR

**Fig. 12:**
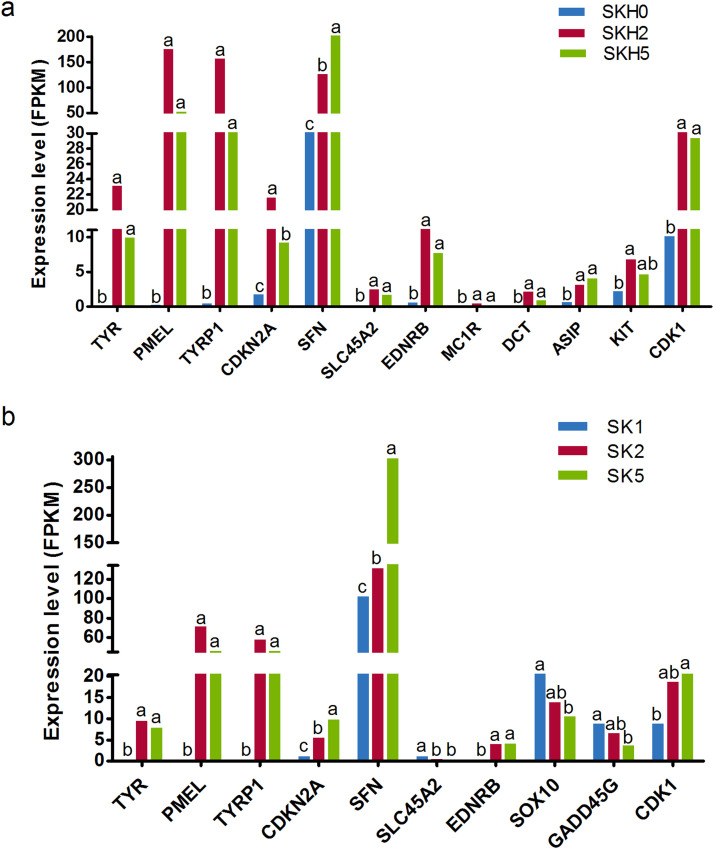
Expression patterns of some candidate genes related to melanin deposition in SHKs (a) and SKs (b).

Among DEGs detected in SKHs and SKs, the expression patterns of some hub genes in PPI networks and well-known genes closely related to melanin deposition, such as TYR, TYRP1, CDKN2A, SFN, PMEL EDNRB, and SLC45A2, were analyzed in detail. In SKHs, TYR, TYRP1, PMEL, EDNRB, SLC45A2, MC1R, DCT, ASIP, KIT and CDK1 showed the same expression patterns, which were upregulated and arrived the peak expression levels at about 14 days of age. The expression levels of CDKN2A were first upregulated and then slightly downregulated across the three stages. SFN showed the extremely high expression levels, and were significantly and continuously upregulated during the formation of the barred feather pattern (P < 0.05) (Fig. 12a). In SKs, the expression patterns of TYR, TYRP1, PMEL, EDNRB and CDK1 were consistent with those in SKHs. And SFN remained high expression levels and continued to increase significantly across the three stages, which showed the same expression patterns with CDKN2A. The expression pattern of SLC45A2 showed different between the back and head skins, although it had low expression levels. In contrast to SKHs, SLC45A2 tended to be downregulated in SKs (Fig. 12b). In addition, MC1R, DCT, ASIP and KIT were found to be differentially expressed in SKHs but not in SKs. SOX10 and GADD45G were found to be differentially expressed in SKs but not in SKHs. These genes may be associated with the different formation processes of barred feather patterns on the head and the back.

**Fig. 13:**
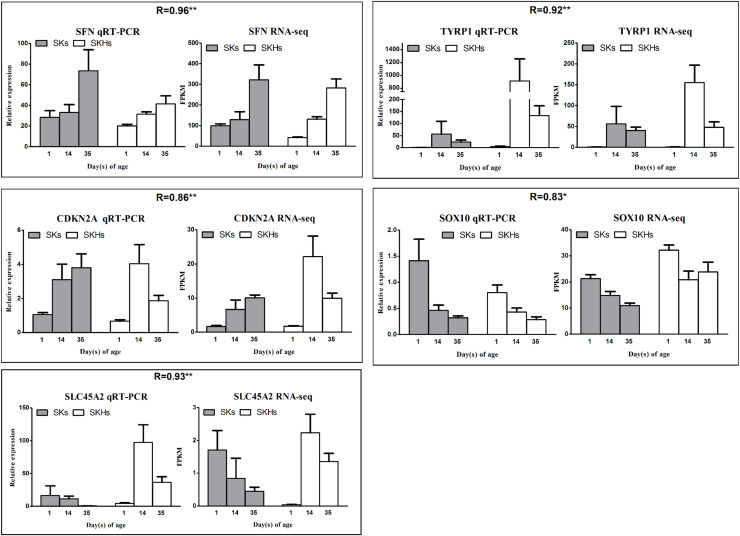
Validation of RNA-seq data by qRT-PCR on five genes. R indicates the Pearson correlation coefficient. ** and * show significance level at p < 0.01 and p < 0.05, respectively.

In order to validate the accuracy of RNA-seq data, five DEGs were selected to performed qPCR in the same samples as RNA-seq, which include two genes with high expression levels (TYRP1 and SFN), two genes with medium expression levels (CDKN2A and SOX10) and one gene with low expression level (SLC45A2). As expected, expression patterns of these genes were in well agreement with the data of RNA-seq (Pearson correlation coefficient: 0.83 ∼ 0.96, P < 0.05) (Fig. 13), demonstrating the reliability of RNA-seq results.

## Discussion

Melanin is the main pigment affecting feather color. The process of melanin deposition is complicated and involves melanocyte development, pigment production and pigment distribution (Cieslak et al., 2011; Muhammad et al., 2025; Ismael et al., 2026). Thus, the development of avian feather color is a complex process involving the combined effects of multiple genes and several signaling pathways (Yang et al., 2020; Wang et al., 2024). At present, the key genes known to be related to melanocyte development include EDNRB (Yang et al., 2020), KIT (Wehrle et al., 2001), SOX10 (Gunnarsson et al., 2011; Zhu et al., 2022) and CDKN2A (Anders et al., 2010). The predominant candidate genes associated with melanin synthesis include TYR (Hasina et al., 2018; Nam et al., 2021), TYRP1 (Yang et al., 2020), DCT (Hasina et al., 2018), MC1R (Yang et al., 2019; Liu et al., 2023), ASIP and MITF (Wang et al., 2022). In addition, PMEL and solute carrier family genes (SLC24A5, SLC45A2, and SLC26A4) are important genes related to chicken feather color and play roles in maintaining the melanosome structure and ensuring the stability of its internal environment (Kerje et al., 2004; Li et al., 2023; Le et al., 2020). The familiar signaling pathways involved in the process of melanin deposition include tyrosine metabolism, melanogenesis, and the MC1R/α-MSH, MAPK/ERK (Jung et al., 2015), PI3K/Akt and Wnt/β-catenin signaling pathways (Pillaiyar et al., 2017; Zhao et al., 2019).

In this study, a typical Chinese sex-linked barred chicken, Wenshang Barred chicken, was selected to explore the key genes and signaling pathways involved in feather pattern formation. Skin follicle tissues from two different body sites were studied to analyze the changes in the transcriptome and regulatory networks of related genes during the formation of the barred feather pattern.

A total of 2291 and 4496 genes were found to have significant (P < 0.05) expression changes in the two body sites of SKs and SKHs, respectively, during the three stages of barred feather formation. The number of DEGs between any two stages in SKHs was much greater than that between the same two stages in SKs (Fig. 3). This might be related to the changes in the feather color on the head being different from those on the back during the formation of the barring pattern. The feathers on the head gradually change from white spots to barring, whereas those on the back change from black to barring. The barring formation process on the head is likely more complex. Despite this, a number of common DEGs were still found between the two tissues in the comparisons of the same two stages (Fig. 4c and d). Among these DEGs, a series of predominant candidate genes related to bird feather color and pattern were included, such as TYR (Hasina et al., 2018; Nam et al., 2021), TYRP1 (Yang et al., 2020), EDNRB (Guo et al., 2022), PMEL (Kerje et al., 2004), SLC45A2, SLC24A5 and SLC26A4 (Li et al., 2023; Le et al., 2020; Guo et al., 2022), indicating that these genes play important roles in feather color formation across the entire body. However, the expression of the KIT, MC1R, ASIP and DCT genes changed in the skin follicle tissues from the top of the head but not in those from the back as the chicks grew from 1 day to 14 days old. Thus, these genes may play a role only in the early stage of feather pattern formation on the head. Interestingly, CDKN2A and CDKN2B were DEGs detected in all comparisons, and they were the common DEGs of the two skin tissues in the comparisons of any two stages. Most importantly, these two genes are located on the sex chromosome Z. Therefore, CDKN2A and CDKN2B may play decisive roles in the pattern formation of sex-linked barred feathers in Wenshang Barred chickens.

Analysis of gene expression profiles at different stages could provide a better understanding of the formation process of plumage patterns. Therefore, we used STEM software to analyze the expression profiles of DEGs in skin tissues from the two body sites (SKs and SKHs). In the back skin, the DEGs were significantly clustered in Profiles 0, 1, 6 and 7 (P < 0.05). Several melanin-related biological processes were enriched in the Profile 6 cluster, such as melanin biosynthetic process, melanosome organization, and melanocyte differentiation, involving the marker genes of melanin deposition processes (TYR, TYRP1 and PMEL) (Yang et al., 2020; Kerje et al., 2004). DEGs clustered in both Profile 7 and Profile 6 were significantly enriched in the cell cycle KEGG pathway. Previous studies have reported that the cell cycle of melanocytes, such as differentiation, apoptosis and arrest, affects the plumage pattern or skin color of animals (Hirobe, 1995; Doreen et al., 2017; Leng et al., 2025). Therefore, it was speculated that barred feather pattern formation is closely related to the cell cycle pathway. DEGs in the skin of the top of the head were also clustered in Profile 6 and Profile 7, as well as Profile 0. Most of the DEGs were significantly enriched in pathways associated with growth and development, reproduction and metabolism. However, KIT, ASIP, and SLC45A2, which are related to melanin production and plumage color, were found in the Profile 6 cluster. The cell cycle-related gene CDKN2B was found in the Profile 7 cluster. Thus, the Profile 7 and Profile 6 clusters may represent important and mainstream expression trends of DEGs during the formation of the barred feather pattern.

A functional enrichment analysis of all the DEGs detected at the two skin sites revealed that 22 and 28 DEGs in the back skin were significantly enriched in melanogenesis and cell cycle pathways, respectively (P < 0.05), and that 38 DEGs in the skin from the top of the head were also significantly enriched in the melanogenesis pathway (P < 0.05). Therefore, melanogenesis and cell cycle pathways may be the key signaling pathways involved in the formation of the plumage pattern of sex-linked barring. In addition, the melanocyte differentiation and melanosome organization GO terms were significantly enriched in the DEGs. Among these DEGs, some well-known feather color-related genes, such as TYR, TYRP1, DCT, PMEL, and KIT, were included. The expression levels and patterns of these genes were analyzed (Fig. 9). The expression levels of TYR, TYRP1 and PMEL increased and then peaked at approximately 14 days of age in both the back and head crown skins, which was consistent with the findings of a previous study in poultry (Wang et al., 2024). These results indicated that TYR, TYRP1 and PMEL play important roles in the early growth and development of black feathers in Wenshang Barred chickens. SFN and CDKN2A were highly expressed in both back and head crown skin. They showed similar expression trends of continuous increase during the formation of barred feathers. Chicken ARF encoded by CDKN2A is able to interact with MDM2 and protect the transcription factor p53 from degradation (Kim et al., 2003). The transcription factor p53 has been showed to activate a number of downstream targets leading to either cell cycle arrest or apoptosis (Doreen et al., 2017). Previous studies have reported that SFN is one of downstream targets of the transcription factor p53 in the cell cycle pathway (Kanehisa et al., 2023). Thus, the RNA expression level of SFN should be indirectly positively regulated by CDKN2A (Fig. 13). The results of our study confirmed the regulatory relationship between them.

PPI regulatory networks of DEGs enriched in melanogenesis and cell cycle pathways, as well as melanin-related biological process GO terms, were constructed in this study. According to multiple algorithms, TYR, TYRP1, CDKN2A and SFN were among the hub genes in the regulatory network (Fig. 11a–d), further indicating that these genes are key genes involved in the regulation of the formation of the barred feather pattern. TYR and TYRP1, which are key rate-limiting enzymes in melanin synthesis (Mani et al., 2001; Westerhof, 2006), may play key roles in the early growth of black feathers and the subsequent black stripe formation of barred feathers through the melanogenesis pathway in Wenshang Barred chickens. SFN, a member of the 14-3-3 gene family, is involved in cell cycle arrest at the G2 phase (Fig. 12, Fig. 13) (Pietenpol et al., 2002). Previous studies have indicated that the white bands are caused by the absence of melanocytes in the feather follicle during the growth of this region (Doreen et al., 2017). Therefore, it is speculated that CDKN2A may indirectly act on the downstream target SFN, inducing cell cycle arrest in melanocytes at the G2 phase (Fig. 13), leading to the temporarily absent of mature melanocytes, thereby causing the formation of white bands in sex-linked barred feathers.

## Conclusion

The formation of sex-linked barred feather patterns in Wenshang Barred chickens is a complex process involving the growth of black plumage and the generation of white bands, with the joint action of multiple genes and multiple signaling pathways. The early growth and development of black feathers, as well as later black stripe formation, are regulated mainly by the melanogenesis pathway and melanocyte differentiation, melanosome organization and melanin biosynthetic biological processes, primarily involving the TYR, TYRP1, EDNRB and PMEL genes. The white bands on the sex-linked barred feathers are associated with regulation of the cell cycle pathway, including the CDKN2A, SFN, CDKN2B, GADD45G and CDK1 genes. In particular, CDKN2A on the Z chromosome and SFN, whose expression is extremely high, may play major roles. It was speculated that SFN may be the downstream target of CDKN2A in the cell cycle pathway, regulating the G2 phase arrest of the melanocyte cycle and causing the formation of white bands. The molecular regulatory processes involved in the formation of barred feathers on the back and head top differ slightly. The different expression patterns of SLC45A2, KIT, MC1R, ASIP, DCT and SOX10 in the two body sites may contribute to the different formation processes of barred feather patterns on the head and the back. These findings provide new insights into the regulation of chicken sex-linked barred feather patterns and provide a valuable theoretical foundation for future breeding selection and related efforts.

## Funding

This work was supported by the National Natural Science Foundation of China (32402749), “JBGS” Project of Seed Industry Revitalization in Jiangsu Province (JBGS [2021] 029), Natural Science Foundation project of Jiangsu Province (BK20221285) and National Key Research and Development Program of China (2021YFD1200803).

## Data availability statement

The raw sequence data reported in this study have been deposited in the Genome Sequence Archive in National Genomics Data Center (NGDC), China National Center for Bioinformation / Beijing Institute of Genomics, Chinese Academy of Sciences (GSA: CRA041310) that are publicly accessible at https://ngdc.cncb.ac.cn/gsa*.*

## CRediT authorship contribution statement

**Qian Xue:** Writing – original draft. **Guohui Li:** Methodology, Conceptualization. **Huiyong Zhang:** Data curation. **Jianmei Yin:** Investigation, Data curation. **Chenghao Zhou:** Software, Formal analysis. **Yixiu Jiang:** Resources, Project administration, Investigation. **Xing Ju:** Visualization, Supervision, Resources. **Wei Han:** Writing – review & editing, Supervision, Funding acquisition.

## Disclosures

The authors declare that they have no conflict of interest.

## Appendix Supplementary materials

Image, application 1

Image, application 2

Image, application 3

Image, application 4

Image, application 5

Image, application 6

Image, application 7

Image, application 8

Image, application 9

Image, application 10

Image, application 11

Image, application 12

## Footnotes

Supplementary material associated with this article can be found, in the online version, at doi:10.1016/j.psj.2026.106945.
