# Supplementary figures and images for: Insights into the mechanism underlying feather pattern formation of sex-linked barring in Chinese native chickens
Source: Poult Sci. 2026 Apr 16;105(7):106945. doi: 10.1016/j.psj.2026.106945 (PMC13191234; doi:10.1016/j.psj.2026.106945)

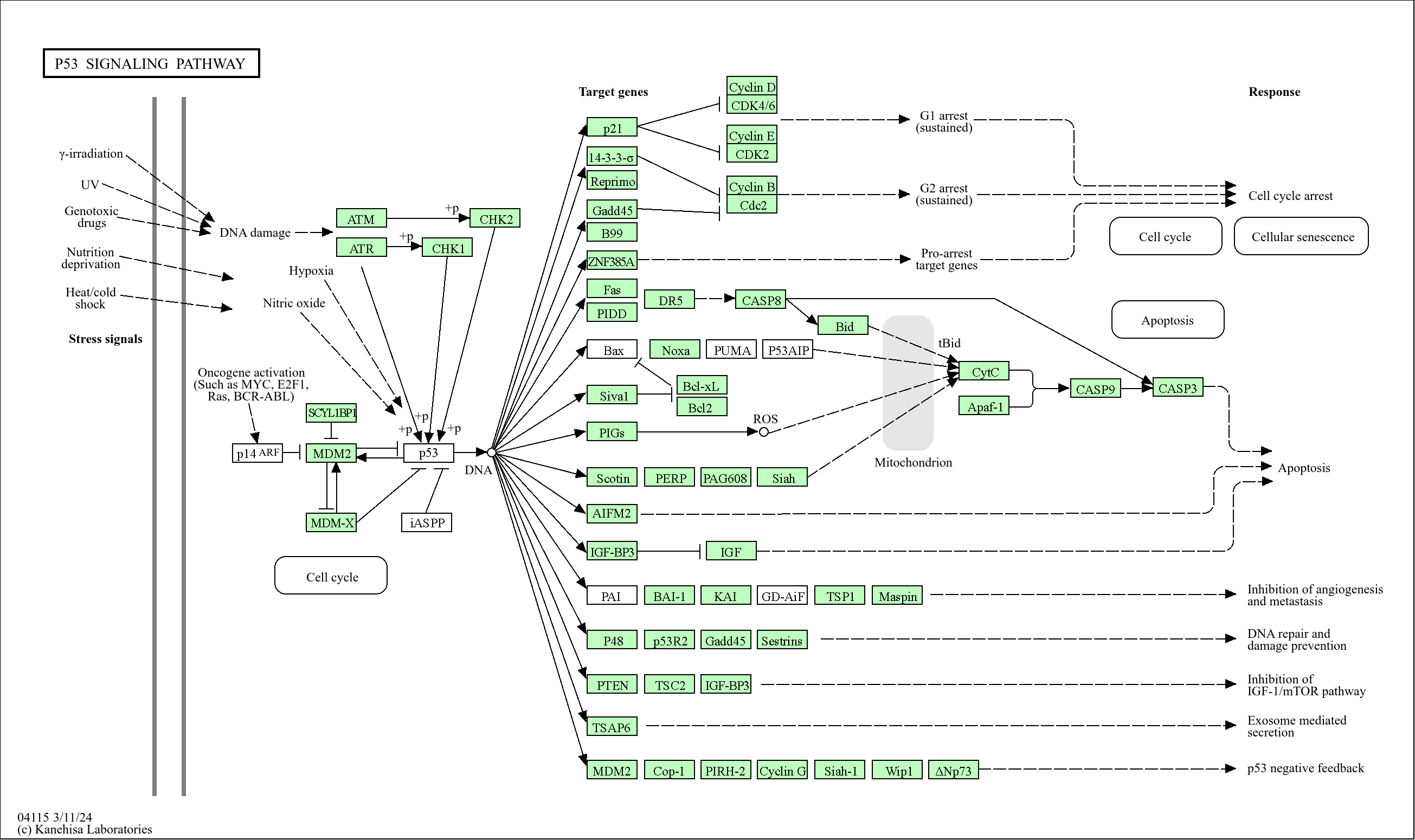

Supplement: Supplementary file 10 [file mmc10.zip › mmc10.png]

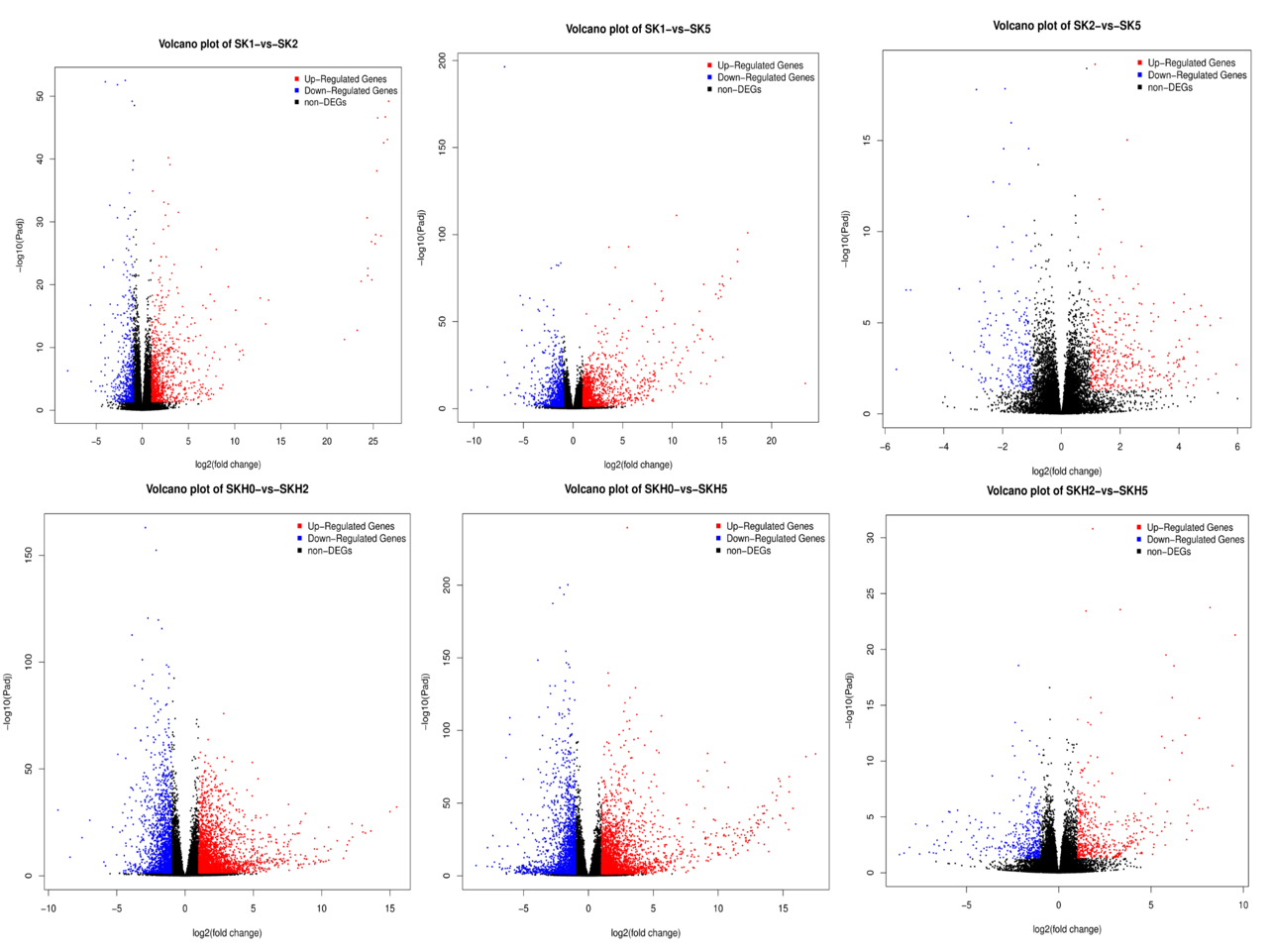

Supplement: Supplementary file 11 [file mmc11.zip › mmc11.png]
